# Microbiome‐Derived Tryptophan Metabolites Regulate AhR Signaling to Restore Epithelial Barrier Integrity in Inflammatory Bowel Disease

**DOI:** 10.1002/mbo3.70386

**Published:** 2026-09-01

**Authors:** Mohammad Ali Mahdiabadi, Asma Moghaddam, Nafiseh Erfanian

**Affiliations:** ^1^ Department of Internal Medicine, School of Medicine Birjand University of Medical Sciences Birjand Iran; ^2^ Cellular and Molecular Research Center Birjand University of Medical Sciences Birjand Iran; ^3^ Department of Anatomy, School of Medicine Birjand University of Medical Sciences Birjand Iran

**Keywords:** aryl hydrocarbon receptor, epithelial barrier, indole metabolites, inflammatory bowel disease, microbiome, postbiotics, tight junctions, tryptophan metabolism

## Abstract

Inflammatory bowel disease (IBD) is characterized by chronic intestinal inflammation and compromised epithelial barrier integrity. Emerging evidence demonstrates that gut microbiota‐derived tryptophan metabolites serve as endogenous ligands for the aryl hydrocarbon receptor (AhR), initiating protective signaling cascades that restore mucosal homeostasis. This review synthesizes current mechanistic insights into how microbial tryptophan catabolites including indole‐3‐aldehyde, indole‐3‐propionic acid, indole‐3‐lactic acid, and indole‐3‐acetic acid activate AhR to enhance epithelial barrier function. However, this protective capacity is specific to activation by physiological, low‐affinity microbial ligands and should not be generalized to AhR signaling irrespective of ligand identity, dose, or duration of exposure. Key bacterial producers include *Lactobacillus* species (*L. reuteri* and *L. plantarum*), *Clostridium sporogenes*, and *Allobaculum* species. AhR activation by these metabolites triggers multiple downstream pathways, including AMP‐activated protein kinase (AMPK) activation, which promotes autophagy and mitochondrial homeostasis; nuclear factor erythroid 2‐related factor 2 (Nrf2)‐mediated antioxidant responses; nuclear factor‐κB (NF‐κB) inhibition, which reduces pro‐inflammatory cytokine production; and interleukin‐22 (IL‐22) induction, which supports epithelial regeneration. These signaling events converge to upregulate tight junction proteins, preserve mucus layer integrity, and reduce actomyosin‐mediated permeability through decreased myosin light chain phosphorylation. Preclinical studies demonstrate AhR‐dependent barrier restoration, with protective effects abolished by AhR antagonists. Despite these preclinical findings, their therapeutic utility in IBD remains to be established in human interventional studies. Importantly, the protective effects of AhR are highly context‐dependent, as kynurenine pathway ligands and sustained receptor activation may exert immunosuppressive or pro‐tumorigenic effects, highlighting the importance of ligand selectivity in therapeutic development.

## Graphical abstract

The graphical abstract illustrates how microbiome‐derived indoles activate the aryl hydrocarbon receptor to restore the intestinal barrier. The picture contrasts the “leaky gut” state with the restored homeostasis achieved through AMPK, Nrf2, and IL‐22 signaling.

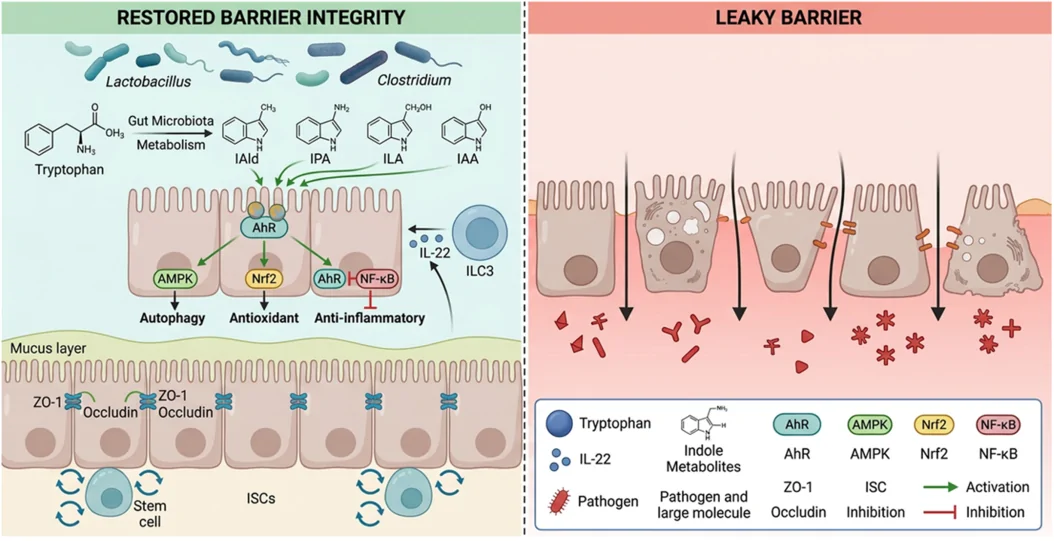

## 1 Introduction

Inflammatory bowel disease (IBD), encompassing Crohn's disease and ulcerative colitis, represents a complex chronic inflammatory disorder of the gastrointestinal tract affecting millions worldwide (Yang et al. 2026). The pathogenesis of IBD involves a multifactorial interplay between genetic susceptibility, environmental factors, immune dysregulation, and alterations in the gut microbiota composition and function (Alemany‐Cosme et al. 2021). A hallmark feature of IBD is the disruption of intestinal epithelial barrier integrity, characterized by increased permeability, compromised tight junction (TJ) architecture, and diminished mucus layer protection (Selvakumar and Samsudin 2025). This barrier dysfunction permits translocation of luminal antigens and microbial products, perpetuating chronic inflammation and tissue damage (Schoultz and Keita 2019).

Recent advances in microbiome research have illuminated the critical role of microbial metabolites as mediators of host–microbiota crosstalk (Wu et al. 2025). Among these, tryptophan‐derived metabolites have emerged as key signaling molecules that interface with host immune and epithelial systems (Barreira‐Silva et al. 2025). The essential amino acid tryptophan serves as a substrate for three major metabolic pathways, such as the kynurenine pathway in host cells, the serotonin pathway in enterochromaffin cells, and the indole pathway in gut bacteria (Haq et al. 2021). Bacterial tryptophan metabolism generates a diverse array of indole derivatives, including indole‐3‐aldehyde (IAld, also referred to in the literature as indole‐3‐carboxaldehyde or indole‐3‐carbaldehyde, a single compound rather than distinct metabolites), indole‐3‐propionic acid (IPA), indole‐3‐lactic acid (ILA), and indole‐3‐acetic acid (IAA), which function as endogenous ligands for the aryl hydrocarbon receptor (AhR) (Miyamoto et al. 2024).

The AhR, originally characterized as a xenobiotic sensor, has been recognized as a crucial regulator of intestinal homeostasis, immune tolerance, and epithelial barrier function (Pernomian et al. 2020). Activation of AhR by microbiota‐derived tryptophan metabolites initiates complex signaling cascades that modulate epithelial cell function, immune cell responses, and barrier integrity (Liu et al. 2026). Zelante et al. (2013) showed that under high dietary tryptophan availability, gut lactobacilli shift to tryptophan catabolism and produce the AhR ligand IAld, driving AhR‐dependent interleukin‐22 (IL‐22) production that balances mucosal reactivity conferring resistance to Candida albicans and protecting the mucosa from inflammatory damage. This established the mechanistic template later extended to TJ regulation, mucus preservation, and epithelial regeneration in subsequent studies.

This review integrates current evidence on microbiome‐derived tryptophan metabolites as key regulators of AhR signaling in intestinal homeostasis and epithelial barrier function in IBD. We highlight their bacterial origins, downstream signaling pathways, and therapeutic potential as postbiotic interventions. We further propose a unifying framework in which these metabolites act as context‐dependent modulators of AhR activity, with effects that range from barrier protection to potential pro‐inflammatory outcomes depending on the microenvironment, providing new insight for microbiome‐targeted therapies in IBD. This paper also highlights the bidirectional relationship between the gut microbiota and intestinal barrier function. While microbiota‐derived indole metabolites promote barrier repair, intestinal inflammation can suppress their production, potentially creating a feedback loop that contributes to disease progression and should be considered in the development of therapeutic strategies.

## 2 Background and Theoretical Foundations

### 2.1 The Gut–Microbiota‐Immune Axis in IBD Pathogenesis

The intestinal mucosa represents the largest interface between the host and the external environment, protecting trillions of commensal microorganisms that collectively constitute the gut microbiota (Fallahi et al. 2025). In healthy individuals, a dynamic equilibrium exists between the microbiota, the intestinal epithelium, and the mucosal immune system, maintaining intestinal homeostasis and preventing inappropriate inflammatory responses. This homeostasis is mediated through multiple mechanisms, including physical barrier function provided by the epithelial monolayer and overlying mucus, antimicrobial peptide secretion, and immune tolerance mechanisms orchestrated by regulatory T cells and tolerogenic dendritic cells (Erfanian et al. 2025; Di Sabatino et al. 2023).

IBD pathogenesis involves a fundamental disruption of this homeostatic balance. Dysbiosis characterized by reduced microbial diversity, depletion of beneficial commensals, and expansion of potentially pathogenic taxa is consistently observed in IBD patients (Lamas et al. 2016). This altered microbial ecosystem exhibits diminished capacity to produce beneficial metabolites, including short‐chain fatty acids and tryptophan derivatives, while potentially generating pro‐inflammatory mediators (Fallahi et al. 2025). Concurrently, genetic susceptibility factors affecting autophagy, pattern recognition receptors, and immune signaling pathways compromise the host's ability to appropriately respond to microbial signals (Lamas et al. 2016).

The epithelial barrier dysfunction observed in IBD represents both a consequence and a driver of chronic inflammation (Turner 2009). Compromised TJ integrity increases paracellular permeability, allowing translocation of bacterial antigens, lipopolysaccharide, and other immunostimulatory molecules into the lamina propria (Kong et al. 2024). This antigenic exposure activates innate immune responses through Toll‐like receptors and NOD‐like receptors, triggering production of pro‐inflammatory cytokines including tumor necrosis factor‐α (TNF‐α), interleukin‐1β (IL‐1β), and interleukin‐6 (IL‐6). These cytokines further compromise barrier function through effects on TJ proteins and actomyosin contractility, establishing a self‐perpetuating cycle of barrier disruption and inflammation (Hooper et al. 2012; K. Saha et al. 2025).

Emerging evidence indicates that the gut–microbiota‐immune axis is critically regulated by microbial metabolites that serve as signaling molecules bridging the microbial and host compartments. Among these, tryptophan‐derived indole metabolites have garnered particular attention due to their capacity to activate the AhR, a transcription factor with pleiotropic effects on epithelial and immune cell function (Miyamoto et al. 2024; Liu et al. 2026). Understanding the mechanisms by which these metabolites restore barrier integrity and modulate immune responses provides a foundation for developing microbiota‐targeted therapeutic strategies for IBD.

### 2.2 The AhR as a Microbiota–Host Interface

The AhR is a ligand‐activated transcription factor belonging to the basic helix–loop–helix/Per‐ARNT‐Sim (bHLH/PAS) family. Initially identified as the mediator of dioxin toxicity, AhR has been recognized as a critical regulator of intestinal homeostasis, with physiological functions extending far beyond xenobiotic metabolism (Perdew 2023). In the intestinal epithelium, AhR is constitutively expressed and serves as a sensor for both dietary and microbiota‐derived ligands, integrating environmental signals to regulate epithelial differentiation, barrier function, and immune responses (Rannug 2020).

In its inactive state, AhR resides in the cytoplasm as part of a multiprotein complex, including heat shock protein 90 (HSP90), AhR‐interacting protein, and p23. Upon ligand binding, AhR undergoes conformational changes leading to nuclear translocation, where it heterodimerizes with the AhR nuclear translocator (ARNT). The AhR–ARNT complex binds to xenobiotic response elements (XREs) or dioxin response elements (DREs) in the promoter regions of target genes, initiating transcription of genes involved in xenobiotic metabolism (cytochrome P450 enzymes), immune regulation, and epithelial homeostasis (Perdew 2023).

In the context of intestinal homeostasis, AhR activation has been demonstrated to promote epithelial barrier integrity, enhance antimicrobial peptide production, regulate intestinal stem cell (ISC) function, and modulate immune cell differentiation and cytokine production (Wisniewski et al. 2021). These beneficial outcomes, however, are not unconditional, the same receptor can drive immunosuppressive, barrier‐impairing, or pro‐tumorigenic programs depending on ligand structure, activation duration, and cellular context.

AhR signaling in intestinal epithelial cells upregulates TJ proteins and maintains the structural integrity of the apical junctional complex (Scott et al. 2020). In immune cells, particularly innate lymphoid cells type 3 (ILC3) and T helper 17 (Th17) cells, AhR activation promotes IL‐22 production, a cytokine critical for epithelial regeneration and antimicrobial defense (Russo et al. 2019). Additionally, AhR signaling has been shown to induce IL‐10 receptor expression in intestinal epithelia, enhancing responsiveness to this key anti‐inflammatory cytokine (Pernomian et al. 2020).

Genetic studies have underscored the importance of AhR in intestinal health. AhR‐deficient mice exhibit increased susceptibility to experimental colitis, impaired epithelial barrier function, and altered immune responses (Scott et al. 2020). Conversely, administration of AhR ligands or interventions that enhance endogenous ligand production confer protection against colitis in multiple experimental models (Zelante et al. 2013; Lamas et al. 2016). These observations have established AhR as a therapeutic target of interest in IBD; however, its utility depends critically on ligand identity and activation context, sustained or high‐affinity AhR engagement by host‐derived kynurenine metabolites can produce immunosuppressive or pro‐tumorigenic rather than barrier‐protective outcomes.

## 3 Microbial Tryptophan Catabolism and Metabolite Profiles

### 3.1 Bacterial Tryptophan Metabolism Pathways

Tryptophan, an essential amino acid obtained through dietary intake, serves as a critical substrate for both host and microbial metabolic pathways. While host cells primarily metabolize tryptophan via the kynurenine pathway (generating kynurenine, kynurenic acid, and quinolinic acid) and the serotonin pathway (producing serotonin and melatonin), gut bacteria employ distinct enzymatic machinery to convert tryptophan into a diverse array of indole derivatives (Agus et al. 2018). These bacterial metabolic pathways represent a major route of tryptophan catabolism in the intestine, with microbial metabolism accounting for a substantial proportion of tryptophan‐derived metabolites in the gut lumen and systemic circulation.

Bacterial tryptophan catabolism is primarily mediated by tryptophanase (TnaA), which converts tryptophan into indole, pyruvate, and ammonia. Indole serves as a central intermediate that is further transformed into a diverse array of bioactive derivatives through oxidation, reduction, and deamination reactions (J. Gao et al. 2018). Both Gram‐positive and Gram‐negative bacteria, including species such as *Escherichia coli*, *Clostridium* spp., and *Lactobacillus*, contribute to this metabolic network (Y. Wang, Moriyama, et al. 2026). These transformations generate key metabolites, including IAld, IAA, ILA, and IPA, whose distinct receptor affinities, microbial sources, and biological functions collectively underlie their context‐dependent effects on intestinal homeostasis (Zelante et al. 2013; Agus et al. 2018).

### 3.2 Key Metabolites and Their Microbial Sources

The landscape of microbiota‐derived tryptophan metabolites encompasses numerous structurally related indole compounds, each with distinct physicochemical properties, receptor binding affinities, and biological activities. Among these, several metabolites have been extensively characterized for their AhR agonist activity and protective effects in experimental colitis models.

*IAld* represents one of the most potent and well‐studied microbial AhR ligands. IAld is produced by anaerobic intestinal bacteria through oxidative metabolism of tryptophan (M. Wang, Guo, et al. 2023). *Lactobacillus reuteri* (*L. reuteri*) has been specifically identified as a key producer of IAld, with this metabolite mediating many of the protective effects of *L. reuteri* in experimental colitis models (Hou et al. 2021).

IAld demonstrates high‐affinity binding to AhR and potent activation of AhR‐dependent transcriptional programs. While M. Wang, Guo, et al. (2023) reported that IAld's protective effect against dextran sulfate sodium (DSS)‐induced colitis is only partially AhR‐dependent, with residual barrier‐restorative activity persisting under partial AhR blockade, D. Shi et al. (2026) found that IAld acted through a more strictly AhR/AMPK‐dependent mechanism with near‐complete loss of efficacy upon AhR inhibition. This discrepancy may reflect differences in the specific indole derivative tested, dosing regimen, or the responsiveness of the cell models used, and highlights that “AhR‐dependence” is not always an all‐or‐none property even for structurally related ligands.

In addition to IAld, structurally related bacterial tryptophan metabolites, including indole‐3‐ethanol and indole‐3‐pyruvate, have also been identified as AhR agonists. These compounds preserve the integrity of the apical junctional complex and associated actin regulatory proteins, including myosin IIA and ezrin, thereby protecting against increased gut permeability in experimental colitis through AhR‐dependent mechanisms (Scott et al. 2020).

*IPA* is another prominent microbial tryptophan metabolite with immunomodulatory and barrier‐protective properties. IPA is produced by specific bacterial taxa, with *Clostridium sporogenes* (*C. sporogenes*) identified as an efficient producer (Ren et al. 2025). IPA functions as an AhR agonist and has been shown to restore TJ proteins and induce IL‐22 production in ulcerative colitis models (Ren et al. 2025). Beyond AhR activation, IPA also modulates the pregnane X receptor (PXR), suggesting multireceptor mechanisms underlying its biological effects. Metabolomic studies have revealed decreased levels of IPA in IBD patients, correlating with disease severity and suggesting that IPA deficiency may contribute to IBD pathogenesis (H. Gao et al. 2025).

*ILA* is produced by *Lactiplantibacillus plantarum* (*L. plantarum*) strains through reductive metabolism of tryptophan. *L. plantarum* DPUL‐S164 has been specifically characterized as a producer of ILA, with this metabolite identified as a key mediator of the probiotic's barrier‐protective effects. ILA activates AhR and induces nuclear factor erythroid 2‐related factor 2 (Nrf2)‐mediated antioxidant responses while simultaneously inhibiting nuclear factor‐κB (NF‐κB) signaling, thereby exerting both barrier‐protective and anti‐inflammatory effects. Administration of *L. plantarum* DPUL‐S164 or purified ILA ameliorates intestinal barrier injury in antibiotic‐treated and DSS‐challenged mice through the AhR/Nrf2/NF‐κB axis (A. Wang, Guan, et al. 2024, 2023).

*IAA* is a tryptophan‐derived metabolite produced by multiple bacterial taxa, including *Lactobacillus* species. IAA functions as an AhR ligand and has been shown to promote IL‐22 production and upregulate TJ protein synthesis through AhR signaling pathways (Ran et al. 2026). Studies in caspase recruitment domain family member 9 (CARD9)‐deficient mice, which exhibit increased susceptibility to colitis, have demonstrated that administration of *Lactobacillus* strains capable of producing IAA and other AhR ligands ameliorates gut inflammation (Lamas et al. 2016). IAA, along with other indole derivatives, represents a key component of the protective metabolite profile generated by tryptophan‐metabolizing commensals.

However, the degree to which IAA's protective effects are genuinely AhR‐mediated has been questioned. Compared with IAld and IPA, IAA exhibits relatively lower binding affinity for AhR, and studies in macrophage models have demonstrated that its anti‐inflammatory effects, including HO‐1 induction and free radical scavenging, are not abolished by AhR antagonism with CH‐223191, suggesting that AhR‐independent mechanisms contribute substantially to IAA's biological activity. These findings indicate that attributing IAA's protective effects exclusively to AhR activation may oversimplify its mechanism of action (Ji et al. 2020) (Table 1).

**Table 1:** Microbiome‐derived tryptophan metabolites and their AhR‐mediated protective effects in IBD.

Metabolite	Model/system	Bacterial source/strain	Target cell/tissue	Disease model	Dose/duration	Major outcome	Reference
Microbiota‐derived AhR ligands	In vivo (mouse) microbiota colonization/transfer	Endogenous production by three *Lactobacillus* strains	Colonic tissue; IL‐22, producing immune cells	CARD9^−^/^−^ colitis; microbiota transfer to germ‐free WT mice	2% (w/v) DSS in drinking water for 7 days, followed by 5 days recovery on plain water	Restored AhR ligand production; attenuated colitis via IL‐22 induction	Lamas et al. (2016)
Indole‐3‐ethanol (IEt), indole‐3‐pyruvate (IPyA), indole‐3‐aldehyde (IAld)	In vitro (Caco‐2 monolayers + TNF‐α challenge) + in vivo (Ahr^+^/^−^ and Ahr^−^/^−^ mice)	Not experimentally isolated in this study; discussion notes these metabolites are produced by gut bacteria, including *Lactobacillus reuteri* and *Clostridium sporogenes*, though human quantification is lacking	Apical junctional complex (tight junctions [TJs]: ZO‐1, occludin; adherens junctions: E‐cadherin, β‐catenin); myosin IIA; ezrin	DSS‐induced colitis (3% w/v, 7 days)	In vitro: 50 μM–1 mM (dose‐dependent) in Caco‐2 cells. In vivo, *Trp‐rich diet:* 42 g Trp/kg diet (vs. 2 g Trp/kg standard chow) for 7 days pretreatment, continued through DSS. In vivo, *individual metabolites:* I3A 1000 mg/kg, IPyA 2900 mg/kg, IEt 600 mg/kg, administered 2 days before DSS induction and continued throughout	Attenuated increased gut permeability (FITC‐dextran, TEER) and AJC disassembly; inhibited MyoIIA and ezrin activation; improved weight loss, colon length, disease activity index, and histopathology; effects *largely but not completely* AhR‐dependent (attenuated, not abolished, in *Ahr^−^/^−^* mice)	Scott et al. (2020)
IAld	In vivo (mouse) + in vitro (Caco‐2, RAW264.7, THP‐1 macrophages)	*Lactobacillus acidophilus, Lactobacillus murinus, L. reuteri, Lactobacillus johnsonii* (endogenous producers; not directly administered)	Colonic tissue; Caco‐2 monolayer; RAW264.7 and THP‐1 macrophages	DSS‐induced colitis (2.5% DSS, 7 days); LPS‐stimulated macrophage inflammation	In vivo: *IAld 20 mg/kg/day orally, 7 days; In vitro: 200 μM, 1–2 h pre‐treatment*	↓IL‐6 (AhR‐dependent via NF‐κB/JNK); ↓IL‐1β and TNF‐α (AhR‐independent); ↓NF‐κB p65 translocation (AhR‐dependent); ↓pMLC/MLCK improving permeability (AhR‐dependent in vitro, partially AhR‐independent in vivo); ↑ZO‐1 (AhR‐dependent), ↑occludin (AhR‐independent); improved TEER and FITC‐dextran permeability	M. Wang, Guo, et al. (2023)
IAld (as metabolite of *L. reuteri* D8)	In vitro (intestinal organoid + LPL co‐culture system) + In vivo (mouse)	*L. reuteri D8 (isolated from pig intestinal lumen; confirmed by 16S RNA sequencing)*	Lamina propria lymphocytes (LPLs/ILC3s) → intestinal organoids/ISCs; jejunum and colon	DSS‐induced colitis (5% DSS, 7 days); TNF‐α‐induced organoid damage (60 ng/mL)	In vivo: 10^8^ CFU/day orally, 27 days; In vitro: IAld 0.1–10 mM; D8: 10^4^ CFU/well, 24 h	↓IL‐6 (AhR‐dependent via NF‐κB/JNK); ↓IL‐1β and TNF‐α (AhR‐independent); ↓NF‐κB p65 translocation (AhR‐dependent); ↓pMLC/MLCK improving permeability (AhR‐dependent in vitro, partially AhR‐independent in vivo); ↑ZO‐1 (AhR‐dependent), ↑occludin (AhR‐independent); improved TEER and FITC‐dextran permeability	Hou et al. (2021)
IAld	In vitro (Caco2 cells + TNF‐α injury model; mouse colonoids) + In vivo (C57BL/6 mouse)	Not strain‐specified	Intestinal epithelial cells (Caco2); mouse colonoids; colonic epithelium/tissue	TNF‐α‐induced Caco2 barrier injury (10 ng/mL, 24 h); DSS‐induced colitis (2.5% DSS in drinking water, 7 days, followed by 3 days regular water; 10‐day total protocol)	In vitro: IAld 1–10 μM, 24 h (2 h for AhR nuclear translocation assay); CH‐223191 (AhR inhibitor) 10 μM; Compound C (AMPK inhibitor) 10 μM; HCQ (autophagy inhibitor) 10 μM. In vivo: IAld 50 mg/kg/day (gavage, from DSS start through day 7); Compound C 1 mg/kg/day (gavage, starting 3 days pre‐DSS)	IAld activated AhR/AMPK signaling, inducing autophagy, preserving mitochondrial function, restoring ZO‐1/occludin, and reducing epithelial permeability. AhR or AMPK inhibition largely abolished these protective effects, indicating near‐complete AhR/AMPK dependence	D. Shi et al. (2026)
Indole‐3‐propionic acid (IPA)	In vivo (WT C57BL/6 mice; Rag1^−^/^−^ mice) + In vitro (human peripheral blood CD4^+^ T cells; Th1/Th17 polarization) + Human (fecal metabolomics/16S rRNA; colon biopsies; PB‐CD4^+^ T cells from IBD patients)	Not administered as bacteria; purified IPA given directly. Endogenous IPA‐producing taxa identified by 16S rRNA as decreased in IBD: *Peptostreptococcus*, *Clostridium*, *Fournierella* (IPA‐consuming taxa increased: *Parabacteroides*, *Erysipelatoclostridium*, *Lachnoclostridium*)	Mucosal CD4^+^ T cells (Th1/Th17); lamina propria mononuclear cells (LPMCs); colonic epithelium/tissue	DSS‐induced acute colitis (2% DSS, 7 days + 3 days water, 10‐day protocol); CD45RBhigh CD4^+^ T‐cell transfer‐induced chronic colitis in Rag1^−^/^−^ mice (8 weeks); human IBD cohort (active CD *n* = 39, remission CD *n* = 26, active UC *n* = 35, remission UC *n* = 33, HC *n* = 59 for clinical/microbiome data; separate cohorts for T‐cell functional assays)	In vivo (acute): 50 mg/kg/day, oral gavage, days 0–10. In vivo (chronic): 50 mg/kg every other day, 8 weeks. In vitro: 100 μM, 2–5 days	IPA‐producing bacteria and fecal IPA decreased in IBD; oral IPA attenuated acute/chronic colitis and Th1/Th17 differentiation. Mechanism: IPA binds HSP70 directly (AhR/PXR‐independent), impairing mitochondrial OXPHOS and activating ASK1/JNK‐caspase apoptotic pathway, selectively inducing Th1/Th17 apoptosis, confirmed by HSPA1A knockdown/overexpression	H. Gao et al. (2025)
Indole‐3‐lactic acid (ILA)	In vivo (Balb/C mice); live versus dead bacteria comparison (S164 vs. D‐S164) versus bacterial Trp‐metabolite extract (S164‐TM) versus purified ILA	*Lactiplantibacillus plantarum* DPUL‐S164 (live S164, heat‐inactivated D‐S164, and its Trp‐metabolite extract S164‐TM containing ILA as predominant component, plus minor I3C and indole‐3‐acetic acid [IAA])	Colonic epithelium (TJs: occludin, claudin1, ZO‐1); goblet cells/mucus layer; serum	Antibiotic cocktail (ABX)‐induced microbiota depletion followed by DSS‐induced ulcerative colitis (2.5% w/v DSS, 7 days) in Balb/C mice	S164/D‐S164: 3 × 10^8^ CFU/0.2 mL, oral, 14 days pretreatment; S164‐TM: 0.2 mL containing 27.264 μg/mL ILA, oral, 14 days; ILA (purified): 27.264 μg/mL, oral, 14 days — all followed by 7‐day DSS exposure	Live S164 (not dead) ameliorated DSS colitis via ILA production; purified ILA reproduced these effects — ↓DAI/inflammation, ↑TJ proteins (occludin/claudin1/ZO‐1), ↑AhR/Cyp1a1/Nrf2/HO‐1, ↓NF‐κB. Serum ILA correlated with all these markers, confirming ILA as a key effector via the AhR–Nrf2–NF‐κB axis	A. Wang, Guan, et al. (2024)
ILA, as component of *L. plantarum* DPUL‐S164 tryptophan metabolite extract (DPUL‐S164‐TM); also I3C and IAA detected as minor components	In vitro HT‐29 cell monolayer; AhR pharmacological inhibition (CH223191) to test dependence	*L. plantarum* DPUL‐S164 (isolated from human feces), cultured in Trp‐supplemented MRS broth; extracellular Trp‐metabolite extract (DPUL‐S164‐TM) tested against MRS‐medium‐only control extract (MRS–TM)	Human colon adenocarcinoma epithelial cells (HT‐29)	LPS‐induced barrier injury (2 μg/mL LPS, cell‐based, noncolitis in vivo model)	DPUL‐S164‐TM/MRS–TM: 1:10 dilution (selected from cytotoxicity screening of 10:1–1:1000), 4 h pretreatment then LPS 20 h; CH223191 (AhR inhibitor): 10 μM, co‐treatment	ILA identified as dominant Trp metabolite in DPUL‐S164‐TM (763× higher than control). Extract ↑AhR/Nrf2/HO‐1 and TJ proteins, ↓NF‐κB/inflammation. AhR blockade abolished Nrf2/TJ effects but NOT NF‐κB inhibition divergent AhR‐dependence across pathways	A. Wang, Guan, et al. (2023)
IAA	In vitro only (RAW264.7 murine macrophage cell line)	Not bacterially administered (pure compound tested)	RAW264.7 macrophages (not intestinal epithelium)	LPS‐induced macrophage inflammation (noncolitis, nonintestinal model)	IAA: 25–1000 μM, 12 h pretreatment + 8–24 h LPS (10 ng/mL) co‐treatment (dose‐ and time‐course tested); SnPP (HO‐1 inhibitor): 10 μM; CH‐223191 (AhR antagonist): 20 μM, 12 h pretreatment	IAA dose‐dependently ↓LPS‐induced inflammatory cytokines (IL‐1β, IL‐6, MCP‐1), NF‐κB activation, and free radicals (NO/ROS); ↑HO‐1. HO‐1 blockade abolished cytokine suppression but not radical scavenging. AhR blockade had no effect on any outcome IAA's protective effects in macrophages are entirely AhR‐independent	Ji et al. (2020)

Abbreviations: AhR, aryl hydrocarbon receptor; AJC, apical junctional complex; AMPK, AMP‐activated protein kinase; ASK1, apoptosis signal‐regulating kinase 1; CD, Crohn's disease; CFU, colony‐forming unit; DAI, disease activity index; DSS, dextran sulfate sodium; FITC, fluorescein isothiocyanate; HCQ, hydroxychloroquine; HT‐29, human colorectal adenocarcinoma cell line; I3C, indole‐3‐carbinol; IAA, indole‐3‐acetic acid; IAld, indole‐3‐aldehyde; IBD, inflammatory bowel disease; IL‐1β, interleukin‐1β; IL‐22, interleukin‐22; IL‐6, interleukin‐6; ILC3, innate lymphoid cells type 3; ISCs, intestinal stem cells; JNK, c‐Jun N‐terminal kinase; LPL, lamina propria lymphocyte; LPS, lipopolysaccharide; MCP‐1, monocyte chemoattractant protein‐1; MLCK, myosin light chain kinase; MRS, deMan, Rogosa, and Sharpe; NF‐κB, nuclear factor‐κB; NO, nitric oxide; Nrf2, nuclear factor erythroid 2‐related factor 2; OXPHOS, oxidative phosphorylation; pMLC, phosphorylation of myosin light chain; PXR, pregnane X receptor; ROS, reactive oxygen species; rRNA, ribosomal RNA; SnPP, tin protoporphyrin IX; TEER, transepithelial electrical resistance; Th17, T helper 17; TM, Trp metabolites; TNF‐α, tumor necrosis factor‐α; Trp, tryptophan; UC, ulcerative colitis; WT, wild type; ZO‐1, zonula occludens‐1.

The bacterial sources of these metabolites reflect specific metabolic capabilities distributed broadly across multiple gut microbial phyla; the following represent well‐characterized examples rather than an exhaustive list, as indole metabolite production is strain‐dependent and extends across a wider range of commensal taxa than the examples below suggest.

- *Lactobacillus species: L. reuteri* (IAld producer) (Hou et al. 2021), *L. plantarum* (ILA producer) (A. Wang, Guan, et al. 2024), *Lactobacillus murinus*, *L. taiwanensis* (AhR‐activating metabolites) (Lamas et al. 2016).
- *Clostridium species: C. sporogenes* (efficient producer of AhR agonists, including IPA) (Rondeau et al. 2024).
- *Allobaculum species:* Producers of AhR‐activating tryptophan metabolites, with abundance decreased in CARD9‐deficient mice (Lamas et al. 2016).
- *E. coli:* Wild‐type strains produce IAld and IPA (Wisniewski et al. 2021).
- *Bifidobacterium species:* Several human gut‐associated *Bifidobacterium* species produce ILA through a tryptophan synthase‐dependent pathway, representing an important source of ILA within the phylum Actinobacteria (Yong et al. 2024; Sinha et al. 2024).
- *Bacteroides species:* Bacteroides ovatus produces the tryptophan‐derived metabolite IAA, which promotes IL‐22 production and contributes to intestinal immune homeostasis, illustrating that tryptophan‐derived indole metabolite production also occurs in members of the phylum Bacteroidetes (Ihekweazu et al. 2021).
- *Ruminococcus gnavus:* Together with *C. sporogenes*, converts tryptophan to tryptamine via tryptophan decarboxylase (Williams et al. 2014); tryptamine itself has been directly characterized as a moderate‐efficacy AhR agonist capable of inducing AhR nuclear translocation and CYP1A1 induction (Vyhlídalová et al. 2020).

This taxonomic distribution spanning Firmicutes, Bacteroidetes, Actinobacteria, and Proteobacteria highlights that indole metabolite production is not confined to a few specialist taxa but represents a broadly distributed metabolic capacity across the gut microbiome, the collective output of which is vulnerable to the dysbiosis characteristic of IBD.

The evidence reviewed above indicates that protective activity is not a uniform property of the indole class as a whole, but rather varies across individual metabolites, doses, and experimental systems. IAld shows consistent barrier‐protective effects across studies, though reported studies differ in the degree of AhR‐dependence. M. Wang, Guo, et al. (2023) reported that IAld's protective effect against DSS‐induced colitis is only partially AhR‐dependent, whereas D. Shi et al. (2026) found a more strictly AhR/AMPK‐dependent mechanism with near‐complete loss of efficacy upon AhR inhibition. Rather than reflecting differences between distinct compounds, this discrepancy likely reflects differences in dosing regimen, treatment duration, or the responsiveness of the specific cell and animal models used, and indicates that “AhR‐dependence” is not a fixed, all‐or‐none property even for a single well‐characterized ligand. IPA engages both AhR and the PXR, complicating attribution of its effects to AhR alone (Ren et al. 2025). ILA's protective activity has been demonstrated using two different *L. plantarum* strains (DPUL‐S164 and Y42) (A. Wang, Guan, et al. 2024, 2023; X. Gao et al. 2026), though this evidence originates from a single research group, leaving independent replication across other laboratories or bacterial sources still lacking. IAA, by contrast, retains its anti‐inflammatory activity even under pharmacological AhR blockade, indicating a substantial AhR‐independent component to its mechanism (Ji et al. 2020). This gradient of mechanistic dependence, suggests that protective potential is best understood as metabolite‐ and context‐specific rather than class‐wide, and that findings under one set of experimental conditions should not be extrapolated to another without direct confirmation.

### 3.3 Dysbiosis and Altered Tryptophan Metabolism in IBD

IBD is consistently associated with gut microbial dysbiosis, characterized by both taxonomic alterations and impaired metabolic capacity (Sultan et al. 2021). Notably, tryptophan metabolism is disrupted, leading to reduced production of AhR‐activating indole derivatives (G. Wang, Fan, et al. 2024). Metabolomic studies have demonstrated decreased levels of key metabolites, including indole‐3‐acetate and indole‐3‐propionate, which correlate with disease activity and inflammatory markers, implicating defective microbial metabolism in IBD pathogenesis.

This deficiency reflects both the depletion of metabolite‐producing taxa such as *Lactobacillus* species, *Clostridium* clusters, and indole‐producing commensals and functional alterations in microbial activity (Lamas et al. 2016; Hou et al. 2021; Rondeau et al. 2024). Host genetic factors further influence this axis; for example, CARD9 deficiency is associated with reduced abundance of *L. reuteri* and Allobaculum species, both known for their capacity to generate AhR‐activating metabolites (Lamas et al. 2016).

Functionally, diminished AhR ligand availability compromises epithelial barrier integrity and immune regulation by limiting IL‐22 production, TJ maintenance, and anti‐inflammatory signaling (Stockinger et al. 2024). Conversely, restoration of microbial tryptophan metabolism through probiotics, postbiotics, or dietary interventions ameliorates experimental colitis via AhR‐dependent mechanisms, highlighting the therapeutic potential of targeting the tryptophan AhR axis in IBD (Sun et al. 2020; De la Rosa González et al. 2024).

Critically, however, this relationship is bidirectional. Active intestinal inflammation itself, through interferon‐γ (IFN‐γ)‐mediated induction of indoleamine 2,3‐dioxygenase 1 (IDO1), diverts available tryptophan away from microbial indole synthesis toward the host kynurenine pathway, thereby further reducing AhR ligand availability when barrier protection is most needed. This creates a self‐amplifying cycle in which reduced microbial indole production and ongoing barrier dysfunction perpetuate one another a bidirectional dynamic that a strictly linear model of the microbiota‐tryptophan‐AhR axis fails to capture (Kiran et al. 2026).

## 4 AhR Signaling Mechanisms in the Intestinal Epithelium

Although the subsequent sections describe the AhR–AMPK, AhR–Nrf2, NF‐κB, and IL‐22 pathways individually for clarity, this sequential presentation should not be mistaken for a linear cascade. These pathways exhibit substantial crosstalk, for instance, Nrf2 activation independently suppresses NF‐κB signaling, and AMP‐activated protein kinase (AMPK) activation can itself induce autophagy‐dependent Nrf2 stabilization such that AhR functions as one node within an interconnected regulatory network rather than the sole upstream trigger of a unidirectional cascade. The bidirectional nature of this axis further underscores that therapeutic strategies must account for network‐level interactions rather than targeting a single linear pathway. Furthermore, before the predominantly protective pathways below are described in detail, it should be noted that this protective characterization applies specifically to activation by microbiota‐derived indoles under the experimental conditions reviewed here, and does not imply that AhR activation is intrinsically or universally beneficial across all ligands, doses, and cellular contexts.

### 4.1 AhR Activation and Receptor Pharmacology

Activation of the AhR by microbiota‐derived tryptophan metabolites initiates signaling cascades that regulate epithelial barrier integrity, immune responses, and metabolic homeostasis. Pharmacological studies using the selective AhR antagonist CH‐223191 demonstrate that the protective effects of indole metabolites are partially AhR‐dependent, as inhibition of the receptor abolishes their barrier‐protective and anti‐inflammatory actions in both in vitro and in vivo models (M. Wang, Guo, et al. 2023). Genetic evidence further supports this dependence, as loss of AhR or disruption of ARNT signaling eliminates the beneficial effects of indole metabolites on epithelial function and cytokine regulation (Stockinger et al. 2021). Among these metabolites, IAld exhibits the highest agonistic potency, while ILA, IPA, and IAA also act as functional AhR ligands with varying efficacy, contributing to differential biological outcomes (Agus et al. 2018).

Importantly, microbial‐derived AhR ligands induce a distinct transcriptional program compared with xenobiotic ligands, such as dioxins. Whereas both activate canonical detoxification pathways, microbiota‐derived metabolites preferentially promote genes involved in barrier maintenance, immune regulation, and epithelial homeostasis (Perdew 2023). This distinction also implies that not all AhR agonists are therapeutically equivalent. Broad or nonselective AhR activation risks reproducing the immunosuppressive transcriptional programs associated with kynurenine‐pathway ligands, a consideration directly relevant to the design of AhR‐targeted postbiotics.

### 4.2 AhR–AMPK Axis: Autophagy and Mitochondrial Homeostasis

AMPK is an indirect downstream effector of AhR signaling rather than a direct transcriptional target of the AhR–ARNT complex; AhR activation by IAld triggers metabolic and mitochondrial adaptations that secondarily engage AMPK phosphorylation, an effect confirmed to be AhR‐dependent by its abolition upon receptor inhibition, but not mediated through direct XRE‐driven transcription of AMPK itself (D. Shi et al. 2026; He and He 2025). Activation of AhR by IAld induces AMPK phosphorylation in intestinal epithelial cells, forming a functional AhR–AMPK axis that enhances barrier integrity, an effect abolished by AhR inhibition, confirming pathway dependence (D. Shi et al. 2026). Pharmacological studies further demonstrate that AMPK activity is required for IAld‐mediated barrier protection (D. Shi et al. 2026). This bidirectional dependency AhR driving AMPK activation, and AMPK being required for full barrier‐protective effect indicates a functional rather than transcriptional coupling between the two pathways. Mechanistically, AMPK activation has been shown to promote autophagy through phosphorylation of ULK1 (Egan et al. 2011) with additional evidence suggesting roles in mitochondrial quality control and metabolic adaptation, though the extent to which these effects are specifically operative downstream of AhR–AMPK signaling in intestinal epithelial cells remains to be directly demonstrated.

Available evidence supports a functional AhR–AMPK axis linking microbial metabolite sensing to autophagy induction and epithelial resilience, though the full mechanistic scope of this coupling particularly regarding mitochondrial homeostasis and metabolic reprogramming requires further direct investigation in IBD‐relevant models (Y. Wang, Huang, Wei, et al. 2026).

### 4.3 AhR–Nrf2 Pathway: Antioxidant Defense

Nrf2 is a master regulator of antioxidant and cytoprotective responses that is activated upon dissociation from Keap1 and nuclear translocation (Tonelli et al. 2018). The relationship between AhR and Nrf2 is not one of direct transcriptional regulation but rather one of convergent signaling crosstalk. Although AhR and Nrf2 share overlapping target genes and can be co‐activated by the same ligands (Kiran et al. 2026), Nrf2 is independently regulated through the canonical Keap1‐dependent redox‐sensing pathway and is not a direct XRE‐driven transcriptional target of AhR (Yamamoto et al. 2018). Therefore, the co‐activation of both pathways by microbial indole metabolites most likely reflects their overlapping yet mechanistically distinct responsiveness to the same ligand, rather than a strictly hierarchical relationship in which AhR acts upstream of Nrf2. For example, ILA activates both AhR and Nrf2, leading to coordinated barrier protection and anti‐inflammatory effects (A. Wang, Guan, et al. 2024). AhR–Nrf2 crosstalk involves transcriptional regulation, shared target genes, and post‐translational modulation. This results in induction of antioxidant enzymes (SOD, catalase, GPx, and HO‐1), enhanced glutathione synthesis, activation of phase II detoxification enzymes, and increased cytoprotective capacity (Köhle and Bock 2007). Together, these effects reduce oxidative stress and epithelial injury, which are key drivers of inflammation in IBD (Turner 2009; Peterson and Artis 2014).

### 4.4 NF‐κB Inhibition and Anti‐Inflammatory Responses

NF‐κB is a central regulator of intestinal inflammation and barrier dysfunction in IBD (Mukherjee et al. 2024). AhR does not suppress NF‐κB through direct transcriptional repression; rather, this inhibition is indirect and operates through at least three intermediary mechanisms, including Nrf2‐mediated antioxidant induction that reduces the oxidative signals driving NF‐κB activation, interleukin‐10 receptor (IL‐10R) upregulation that amplifies STAT3‐dependent anti‐inflammatory signaling, and physical interference between the AhR–ARNT complex and NF‐κB transcriptional machinery at shared promoter regions (Liu et al. 2026; Mukherjee et al. 2024). This inhibition also involves modulation of upstream signaling (IKKs/IκB) and induction of anti‐inflammatory mediators. Functionally, this leads to decreased pro‐inflammatory cytokines (TNF‐α, IL‐1β, and IL‐6), reduced chemokine production, preservation of TJ integrity, and protection against epithelial apoptosis (S. Saha et al. 2024). Overall, this axis restores intestinal homeostasis by simultaneously suppressing inflammation and preserving barrier function.

### 4.5 IL‐22 Production and Epithelial Regeneration

IL‐22 is a key epithelial‐protective cytokine produced by ILC3 and Th cells that promotes barrier repair via STAT3 activation. Unlike AMPK and NF‐κB suppression, which are indirect consequences of AhR activation, IL‐22 induction represents a more direct transcriptional effect. AhR–ARNT complexes bind to regulatory elements in the IL‐22 promoter in ILC3 and Th17 cells, making IL‐22 one of the more proximal and well‐validated direct targets of AhR in the intestinal immune compartment (Russo et al. 2019; Sonnenberg et al. 2010). Microbial tryptophan metabolites enhance IL‐22 production through AhR activation in immune cells, particularly ILC3, as shown for IAld from *L. reuteri* (Hou et al. 2021). M. Ma et al. (2023) showed that urolithin A indirectly boosted IL‐22 levels by remodeling gut microbiota composition and restoring endogenous tryptophan metabolism, whereas Han et al. (2025) achieved a comparable increase in IL‐22 and mucosal healing by directly administering postbiotic metabolite preparations, bypassing the need for microbiota remodeling altogether. The convergence of these two distinct strategies indirect microbiota modulation versus direct metabolite delivery on the same AhR–IL‐22 outcome strengthens, rather than merely illustrates, the centrality of this axis in mucosal healing.

### 4.6 IL‐10 Receptor Expression and Anti‐Inflammatory Signaling

IL‐10 is a key anti‐inflammatory cytokine signaling through IL‐10R–STAT3 to maintain intestinal immune homeostasis (P. Murray 2006). IL‐10R1 induction similarly represents a relatively direct transcriptional consequence of AhR activation in intestinal epithelial cells, supported by promoter‐binding evidence. However, the downstream anti‐inflammatory effect amplified IL‐10 signaling is indirect, requiring the presence of sufficient extracellular IL‐10, and therefore depends on the concurrent availability of IL‐10‐producing immune cells in the mucosal environment (Lanis et al. 2017).

AhR activation induces IL‐10R1 expression in intestinal epithelial cells, a mechanism demonstrated using the host‐derived tryptophan metabolite kynurenine (Lanis et al. 2017); whether microbial indole metabolites engage this same regulatory element remains to be directly tested. Where operative, this induction would increase epithelial sensitivity to IL‐10, amplifying STAT3‐mediated anti‐inflammatory signaling, suppressing pro‐inflammatory mediators, and promoting immune‐epithelial homeostasis, representing an indirect but potentially important amplification of endogenous anti‐inflammatory pathways.

Collectively, these pathways do not operate independently but form an integrated signaling network in which AhR activation coordinates metabolic adaptation (AMPK), redox balance (Nrf2), inflammatory suppression (NF‐κB), and regenerative signaling (IL‐22), thereby orchestrating a unified epithelial‐protective response (Table 2).

**Table 2:** Major AhR‐associated signaling pathways relevant to intestinal barrier regulation in IBD.

Signaling pathway	Mechanism of activation	Major cellular effects	Functional outcome in IBD	Representative study (model/system)
Cytoskeletal regulation	AhR signaling decreases MLCK activity and MLC phosphorylation	Reduced actomyosin contraction and junctional tension	Decreased intestinal permeability (“leaky gut”)	Scott et al. (2020) in vivo mouse colitis model
AhR–IL‐22 axis	AhR activation in ILC3 and Th17 cells stimulates IL‐22 secretion	Enhanced intestinal stem cell regeneration, epithelial proliferation, antimicrobial peptide expression, and goblet‐cell support	Accelerated mucosal healing and restoration of epithelial barrier integrity	Hou et al. (2021) intestinal organoid‐LPL co‐culture and DSS‐induced mouse colitis
AhR–AMPK axis	AhR activation induces AMPK phosphorylation	Enhanced AMPK‐dependent autophagy and cellular energy adaptation	Improved epithelial barrier stability and tight junction (TJ) preservation	D. Shi et al. (2026) in vitro TNF‐α‐injured IEC + in vivo DSS‐mouse
AhR–Nrf2 pathway	AhR–Nrf2 signaling crosstalk and antioxidant gene induction	Increased expression of HO‐1, SOD, CAT, and GSH‐related antioxidant enzymes	Reduced oxidative stress and epithelial injury	A. Wang, Guan, et al. (2023) in vitro HT‐29 monolayer; A. Wang, Guan, et al. (2024) in vivo DSS/antibiotic mouse
NF‐κB inhibition	AhR suppresses NF‐κB transcriptional activity and NF‐κB‐dependent inflammatory gene transcription	Reduced TNF‐α, IL‐1β, IL‐6 production	Attenuation of intestinal inflammation and barrier damage	A. Wang, Guan, et al. (2023) in vitro HT‐29 monolayer; A. Wang, Guan, et al. (2024) in vivo DSS/antibiotic mouse
TJ regulation	AhR‐mediated preservation and upregulation of TJ proteins	Increased ZO‐1, occludin, claudins	Improved epithelial barrier integrit of epithelial barrier integrity	A. Wang, Guan, et al. (2024, 2023) and X. Gao et al. (2026)
AhR–IL‐10R signaling	AhR directly induces IL‐10 receptor (IL‐10R1) transcription in intestinal epithelial cells	Increased epithelial responsiveness to IL‐10‐mediated anti‐inflammatory signaling	Maintenance of immune tolerance and epithelial homeostasis	Lanis et al. (2017) in vitro IEC luciferase‐based IL‐10R1 promoter assay + in vivo DSS‐mouse; note: ligand used was the host‐derived kynurenine (produced via IFN‐γ/IDO1 induction), not a microbiota‐derived indole metabolite

Abbreviations: AhR, aryl hydrocarbon receptor; AMPK, AMP‐activated protein kinase; CAT, catalase; DSS, dextran sulfate sodium; GSH, glutathione; HO‐1, heme oxygenase‐1; IEC, intestinal epithelial cell; IFN‐γ, interferon‐γ; IL‐10, interleukin‐10; IL‐10R, interleukin‐10 receptor; IL‐22, interleukin‐22; ILC3, innate lymphoid cells type 3; LPL, lamina propria lymphocyte; MLC, myosin light chain; MLCK, myosin light chain kinase; NF‐κB, nuclear factor‐κB; Nrf2, nuclear factor erythroid 2‐related factor 2; SOD, superoxide dismutase; Th17, T helper 17; TNF‐α, tumor necrosis factor‐α; ZO‐1, zonula occludens‐1.

### 4.7 Context‐Dependent Dual Role of AhR Signaling in IBD

The AhR functions as a critical environmental sensor that integrates diverse microbial, dietary, and host‐derived signals to maintain intestinal homeostasis. Its role in IBD is characterized by a genuine duality wherein AhR activation can promote mucosal healing and barrier protection, or, depending on context, contribute to immune dysregulation and disease progression (Ran et al. 2026; Y. Chen et al. 2023). This duality is not arbitrary but is determined by at least three convergent factors, including ligand structure and binding affinity, the duration and magnitude of receptor engagement, and the cell‐type‐specific transcriptional landscape in which activation occurs.

First, ligand identity is a primary determinant of outcome. Because AhR is a ligand‐activated transcription factor, different ligands induce distinct conformational changes that dictate which co‐factors are recruited and which downstream gene subsets are activated (X. Ma, Chen, et al. 2025). Microbial indole metabolites (IAld, IPA, ILA, and IAA) are comparatively weak, rapidly metabolized agonists that produce transient, low‐amplitude AhR activation compatible with barrier maintenance and IL‐22‐driven repair (Moutusy and Ohsako 2024). By contrast, host‐derived kynurenine pathway metabolites, generated via IDO1/TDO during chronic inflammation, act as AhR ligands that drive sustained receptor engagement associated with Treg induction, suppression of effector T‐cell and NK‐cell function, and, in tumor microenvironments, immune evasion (Mezrich et al. 2010; Opitz et al. 2011). This indicates that microbial and host‐derived AhR ligands are mechanistically distinct signals that are not functionally interchangeable despite converging on the same receptor (I. A. Murray et al. 2014).

Second, the duration and magnitude of AhR activation shape the biological output. Brief, low‐level engagement by physiological ligands supports epithelial homeostasis, whereas sustained, high‐affinity activation exemplified by the prototypic toxicant TCDD overwhelms the receptor's normal proteasome‐mediated negative feedback and impairs epithelial barrier integrity (Y. Chen et al. 2023). This indicates that AhR operates within a narrow physiological window, in which both insufficient and excessive activation are associated with intestinal pathology.

Third, cell‐type‐specific transcriptional programs further diversify AhR signaling outcomes. The same ligand can induce IL‐22 production in ILC3s and Th cells while engaging distinct protective or tolerogenic programs in intestinal epithelial cells, dendritic cells, and macrophages (Z. Ma, Wang, et al. 2025; J. Chen et al. 2020). This cell‐intrinsic heterogeneity means that the net physiological effect of AhR activation reflects the integrated response across multiple cell populations rather than a single uniform output, and helps explain why pharmacological AhR agonists with differing receptor residence times and tissue distributions such as rationally designed heterocyclic agonists can be engineered to selectively favor tissue‐repair programs over immunosuppressive ones (J. Chen et al. 2020).

A further contradictory finding concerns AhR's relationship with Th17 differentiation. Studies in autoimmune models have demonstrated that AhR activation by the high‐affinity tryptophan‐derived ligand 6‐formylindolo[3,2‐b]carbazole (FICZ) enhances IL‐17 expression and Th17 differentiation, an outcome that could amplify rather than suppress intestinal inflammation if replicated by therapeutic AhR agonists (Quintana et al. 2008). Whether microbiota‐derived indole metabolites, which engage AhR with lower affinity and shorter residence time, carry the same risk remains incompletely characterized and represents an important gap in the current literature.

## 5 Restoration of Epithelial Barrier Function

### 5.1 TJ Protein Regulation

The intestinal epithelial barrier is primarily maintained by TJ complexes composed of claudins, occludin, and ZO proteins. In IBD, inflammatory cytokines, oxidative stress, and actomyosin contraction disrupt TJ integrity, leading to increased permeability (“leaky gut”) (K. Saha et al. 2025). Several microbial tryptophan metabolites, including IAld, ILA, and IPA, have been shown to improve barrier function by upregulating TJ proteins through predominantly AhR‐dependent mechanisms, though the degree of AhR‐dependence varies between metabolites and the evidence for IAA specifically involves AhR‐independent pathways (A. Wang, Guan, et al. 2024; He and He 2025).

Specifically, they increase ZO‐1, occludin, and claudin expression, preserve apical junctional complex architecture, and maintain cytoskeletal linkages (e.g., myosin IIA and ezrin), thereby preventing junctional disassembly (D. Shi et al. 2026; Su et al. 2022). Mechanistically, these effects involve AhR‐driven transcriptional regulation, AMPK‐mediated junction stabilization, and Nrf2‐dependent antioxidant protection, with NF‐κB inhibition providing additional protection against inflammation‐induced TJ loss. Collectively, these changes translate into measurable reductions in epithelial permeability and improvements in barrier integrity in preclinical colitis models, though the degree of functional restoration and its durability under chronic inflammatory conditions have not been systematically characterized (Rothhammer and Quintana 2019).

### 5.2 Mucus Layer Preservation and Goblet‐Cell Function

The mucus layer, primarily composed of mucin 2 (MUC2) from goblet cells, forms the first physical barrier separating microbiota from epithelium. In IBD, mucus depletion, altered secretion, and increased degradation weaken this protective layer (Gustafsson and Johansson 2022).

Microbial tryptophan metabolites preserve mucus integrity through the AhR–IL‐22 axis (Kang et al. 2025). IL‐22 enhances goblet‐cell function, promotes mucus production, and induces antimicrobial peptide expression, thereby reinforcing mucosal barrier defense strengthening mucus defense (Keir et al. 2020). Additionally, AhR signaling influences goblet‐cell differentiation (e.g., via FICZ and MAPK/ERK pathways), contributing to long‐term restoration of mucus‐producing cell populations (Wisniewski et al. 2021). Overall, these effects reinforce both structural and functional integrity of the mucus barrier.

### 5.3 Actomyosin Regulation and Permeability Control

Epithelial permeability is dynamically regulated by the actomyosin cytoskeleton via myosin light chain kinase (MLCK)‐mediated myosin light chain (MLC) phosphorylation. In IBD, inflammatory cytokines (TNF‐α and IFN‐γ) increase phosphorylation of myosin light chain (pMLC), driving actomyosin contraction and TJ opening (Al‐Sadi 2009). Microbial tryptophan metabolites counteract this process by reducing pMLC levels through AhR‐dependent signaling. This leads to reduced MLCK activity, stabilization of actin cytoskeleton (myosin IIA and ezrin) and decreased junctional tension. These changes result in reduced paracellular permeability and improved barrier function in both in vivo colitis and epithelial cell models (Rothhammer and Quintana 2019; Hu et al. 2022).

### 5.4 ISC Homeostasis

**Figure 1:**
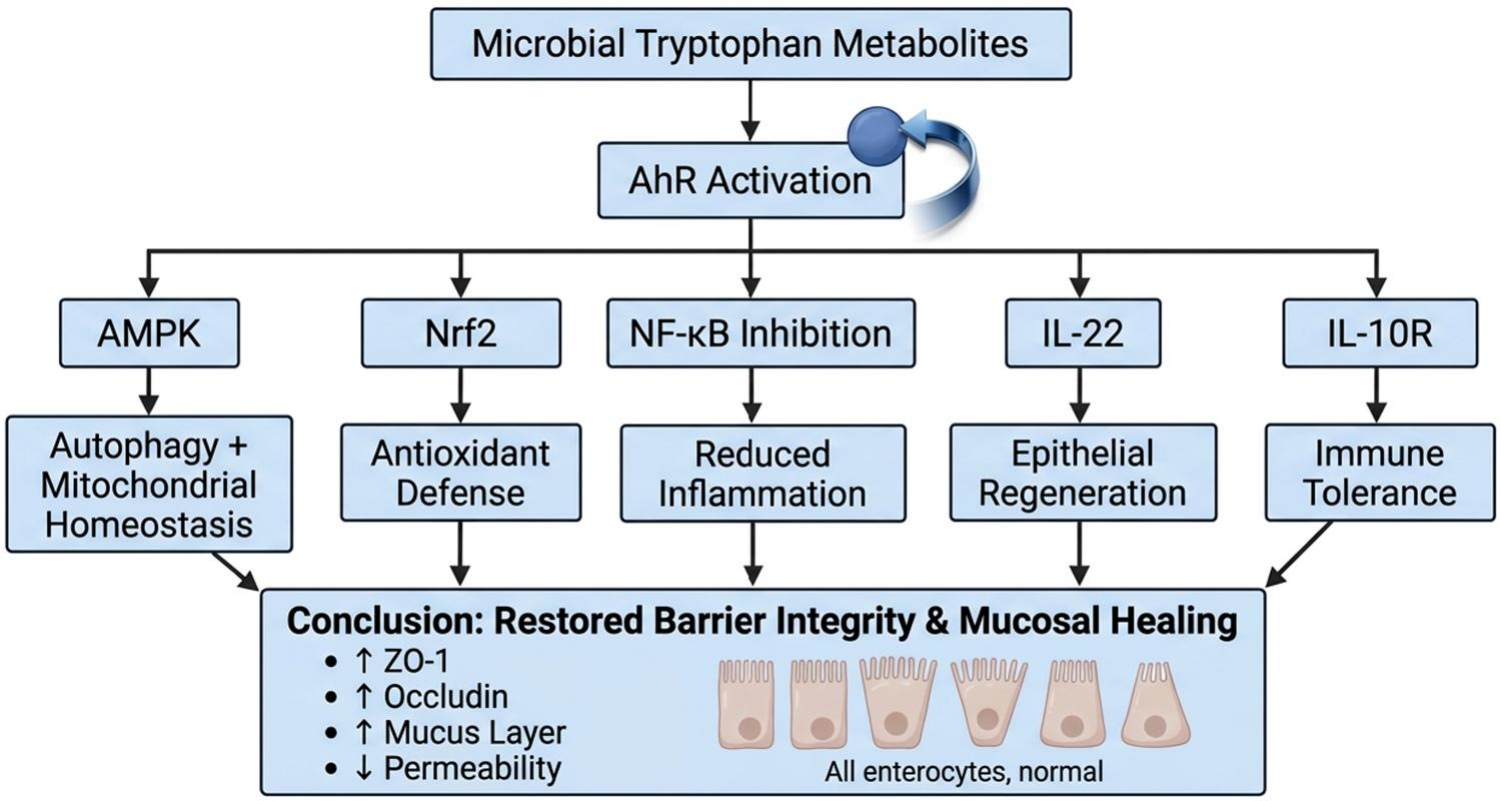
Gut bacterial metabolites derived from tryptophan, including IAld, IPA, ILA, and IAA, activate AhR signaling and downstream pathways, such as AMPK, Nrf2, NF‐κB, and IL‐22. These coordinated responses enhance tight junction integrity, antioxidant defense, mucus production, and epithelial regeneration. Conversely, active inflammation suppresses indole‐producing taxa and diverts tryptophan toward the host kynurenine pathway via IDO1 induction, illustrating that this axis operates as a bidirectional feedback loop rather than a one‐way signaling cascade. AhR, aryl hydrocarbon receptor; AMPK, AMP‐activated protein kinase; IAA, indole‐3‐acetic acid; IAld, indole‐3‐aldehyde; IDO1, indoleamine 2,3‐dioxygenase 1; IL‐22, interleukin‐22; ILA, indole‐3‐lactic acid; IPA, indole‐3‐propionic acid; NF‐κB, nuclear factor‐κB; Nrf2, nuclear factor erythroid 2‐related factor 2; ZO‐1, zonula occludens‐1.

ISCs maintain continuous epithelial renewal and are essential for mucosal repair. In IBD, inflammation and oxidative stress impair ISC function and regenerative capacity. Microbial tryptophan metabolites support ISC homeostasis through the AhR–IL‐22–STAT3 axis, with IL‐22 specifically enhancing ISC proliferation and epithelial regeneration (Lindemans et al. 2015). In addition, organoid studies demonstrate direct enhancement of growth and survival under inflammatory stress. AhR signaling also regulates ISC fate decisions (stemness vs. differentiation), influencing epithelial lineage composition (e.g., goblet cells via FICZ–ERK–Notch modulation). Furthermore, AMPK and Nrf2 pathways protect ISCs from metabolic and oxidative stress, preserving the regenerative pool. Overall, these effects sustain long‐term epithelial renewal and mucosal healing (Lindemans et al. 2015; Metidji et al. 2018; Sato et al. 2011) (Figure 1).

## 6 Preclinical and Clinical Evidence

### 6.1 DSS‐Colitis Models and Mechanistic Validation

The DSS‐induced colitis model is widely used to investigate IBD pathogenesis and evaluate therapeutic strategies. DSS induces epithelial barrier disruption, immune activation, and inflammatory cytokine production, closely mimicking key features of human colitis (Chassaing et al. 2014).

Scott et al. (2020) demonstrated AhR‐dependent protection focused primarily on cytoskeletal stabilization of the apical junctional complex, whereas Zelante et al. (2013) identified IL‐22 induction as the principal protective mechanism. These complementary rather than identical findings suggest that AhR‐dependent protection against DSS‐induced colitis is mediated through multiple, partially nonoverlapping downstream effector arms rather than a single unified pathway. IAld significantly ameliorates disease severity and preserves epithelial barrier integrity in DSS models (Puccetti et al. 2023), consistent with the strict AhR‐dependence established pharmacologically in Section 4.1.

ILA derived from *L. plantarum* and IPA both improve barrier function and reduce inflammation via AhR/Nrf2 activation and IL‐22 induction. These metabolites enhance TJ integrity and suppress pro‐inflammatory signaling (A. Wang, Guan, et al. 2023; X. Gao et al. 2026).

Microbiota‐targeted interventions further support this axis. Compounds such as Ganoderic acid A, berberine, and traditional Chinese medicine formulations modulate gut microbial tryptophan metabolism, increasing production of AhR ligands and promoting IL‐22‐mediated mucosal repair (Kou et al. 2024; X. Wang, Huang, Zhang, et al. 2023; Jing et al. 2021). Postbiotic preparations similarly recapitulate protective effects by directly delivering bioactive metabolites (Han et al. 2025). Mechanistic validation across independent approaches supports strict AhR‐dependence. Pharmacological blockade with the antagonist CH‐223191 attenuated the barrier‐protective effects of IAld (M. Wang, Guo, et al. 2023), while in a separate model, AhR signaling through the MK2/p‐MK2/TTP pathway was shown to mediate anti‐inflammatory effects in DSS‐induced colitis (Q. Wang et al. 2018; Zhao et al. 2010). Together, these pharmacological and pathway‐level findings support AhR‐dependence, though direct genetic knockout/knockdown evidence specific to these barrier‐protective effects would further strengthen causal certainty. Table 3 summarizes the evidence levels for the key mechanistic and therapeutic claims reviewed in this article, distinguishing experimental, correlational, and hypothetical findings.

**Table 3:** Evidence levels for key mechanistic and therapeutic claims in this review.

Claim	Evidence type	Experimental model	Evidence strength
IAld protects against colitis via AhR	Experimental	DSS‐colitis mouse + in vitro epithelial cells	Strong preclinical; AhR‐dependence partially supported by CH‐223191 antagonism (M. Wang, Guo, et al. 2023)
ILA ameliorates barrier injury via AhR/Nrf2/NF‐κB	Experimental	DSS‐colitis and antibiotic‐treated mouse models	Moderate preclinical; single research group (A. Wang, Guan, et al. 2024, 2023)
IPA restores tight junction proteins and induces IL‐22	Experimental	Ulcerative colitis murine models	Moderate preclinical; multireceptor (AhR + PXR) complicates attribution (H. Gao et al. 2025)
Microbiota‐derived AhR ligands promote IL‐22‐mediated protection against colitis	Experimental	CARD9‐deficient mouse model; microbiota transfer; human IBD fecal samples	Strong preclinical + human correlative; CARD9 deficiency impairs microbial AhR ligand production, reducing IL‐22; protection restored by AhR ligand‐producing *Lactobacillus* strains or AhR agonist (Lamas et al. 2016)
AhR → AMPK axis enhances barrier integrity	Experimental	In vivo murine model + in vitro epithelial cells	Moderate preclinical; indirect functional coupling; mechanism not fully resolved at transcriptional level (D. Shi et al. 2026; He and He 2025)
AhR–Nrf2 crosstalk reduces oxidative stress	Experimental	In vitro epithelial cells + murine models	Moderate; convergent co‐activation, not hierarchical AhR → Nrf2 relationship (A. Wang, Guan, et al. 2024, 2023; Köhle and Bock 2007)
AhR suppresses NF‐κB via indirect mechanisms	Experimental	In vitro and DSS‐colitis models	Moderate; indirect pathway via Nrf2, IL‐10R, and promoter interference; mechanism not fully resolved (Liu et al. 2026; Mukherjee et al. 2024)
AhR → IL‐22 induction in ILC3/Th17 cells	Experimental	Murine models + in vitro immune cells	Strong preclinical; direct transcriptional target of AhR–ARNT complex (Russo et al. 2019; Sonnenberg et al. 2010)
AhR upregulates IL‐10R1 in epithelial cells	Experimental	In vitro IEC (luciferase‐based IL‐10R1 promoter assay) + in vivo DSS‐colitis mouse	Moderate; direct evidence (Lanis et al. 2017); but uses host‐derived kynurenine, not a microbial indole extrapolation untested; downstream effect indirect, IL‐10‐dependent
Kynurenine pathway AhR ligands → immunosuppression	Experimental	In vitro immune cells; tumor microenvironment models	Moderate; context‐dependent; demonstrated in Treg induction and tumor models, not in IBD‐specific models (Mezrich et al. 2010; Opitz et al. 2011)
Indole metabolites depleted in IBD patients	Correlational	Human cross‐sectional metabolomic studies	Associative only; causation not established; methodological heterogeneity across studies (Franzosa et al. 2019; Lavelle and Sokol 2020)
Microbial dysbiosis reduces AhR ligand availability in IBD	Correlational	Human observational + genetic (CARD9) studies	Associative; confounded by diet, medication, and disease stage (Lamas et al. 2016; Kiran et al. 2026)
Postbiotic indole metabolites as IBD therapeutics	Hypothetical	No human RCT available	Biologically plausible; clinically unproven (Maftei et al. 2026)
Probiotic strains restore tryptophan‐AhR axis in IBD	Hypothetical	Murine probiotic intervention studies only	Promising preclinical; no human interventional data (De la Rosa González et al. 2024; Maftei et al. 2026)

Abbreviations: AhR, aryl hydrocarbon receptor; AMPK, AMP‐activated protein kinase; DSS, dextran sulfate sodium; IAld, indole‐3‐aldehyde; IBD, inflammatory bowel disease; IEC, intestinal epithelial cell; IL‐10R, interleukin‐10 receptor; IL‐22, interleukin‐22; ILA, indole‐3‐lactic acid; IPA, indole‐3‐propionic acid; NF‐κB, nuclear factor‐κB; Nrf2, nuclear factor erythroid 2‐related factor 2; PXR, pregnane X receptor; RCT, randomized controlled trial.

Despite its widespread use, the DSS‐colitis model has important translational limitations. It induces acute chemical epithelial injury rather than the chronic, relapsing‐remitting, immune‐mediated pathology of human IBD, and metabolite doses used in murine studies frequently exceed physiologically achievable intestinal concentrations in humans. These considerations do not invalidate the mechanistic insights gained from this model, but indicate that preclinical efficacy should be regarded as hypothesis‐generating rather than predictive of clinical outcomes (Chassaing et al. 2014; Perše 2026).

### 6.2 Probiotic and Postbiotic Interventions

Therapeutic strategies targeting microbial tryptophan metabolism include probiotic, postbiotic, and microbiota‐modulating approaches. Probiotic interventions using tryptophan‐metabolizing bacteria such as *L. reuteri* (A. Wang, Guan, et al. 2024), *L. plantarum* (J. Shi et al. 2020), and *C. sporogenes* (Rondeau et al. 2024) restore AhR ligand availability, leading to improved epithelial barrier integrity and reduced inflammation. These effects are particularly evident in dysbiosis‐associated models, such as CARD9 deficiency, where restoration of microbial tryptophan metabolism corrects impaired AhR signaling (Lamas et al. 2016). Postbiotic approaches, based on purified metabolites such as IAld, ILA, and IPA, reproduce the beneficial effects of live bacteria. These compounds directly activate AhR signaling, enhance IL‐22 production, restore TJ integrity, and reduce inflammatory responses in colitis models (Venkatesh et al. 2014).

Compared with probiotics, postbiotics offer advantages including dose standardization, improved stability, reduced safety concerns, and more direct mechanistic targeting. Additionally, pharmacological and dietary interventions can indirectly enhance endogenous production of tryptophan‐derived AhR ligands, offering alternative strategies for microbiota modulation. Overall, both approaches join in the restoration of the tryptophan‐AhR–IL‐22 axis as a central mechanism of mucosal protection in preclinical settings (Maftei et al. 2026). However, it should be emphasized that neither probiotic nor postbiotic strategies targeting this axis have been evaluated in randomized human trials, and the translation of these findings to clinical practice will require demonstration of efficacy, safety, and pharmacokinetic adequacy in IBD patients.

### 6.3 Translational Potential for Human IBD

Human studies provide supportive but still limited translational evidence. Metabolomic analyses consistently demonstrate reduced levels of microbial‐derived indole metabolites in IBD patients, correlating with disease severity and inflammatory markers. This translational gap is further compounded by the fact that the majority of mechanistic evidence reviewed in preceding sections derives from DSS‐induced colitis an acute chemical injury model rather than from models that more closely replicate the chronic immune‐mediated pathology of human IBD, such as IL‐10‐knockout, T‐cell transfer colitis, or CARD9‐deficient models.

Franzosa et al. (2019) reported reduced fecal indole‐3‐acetate and indole‐3‐propionate in IBD patients using untargeted LC‐MS metabolomics, while Lavelle and Sokol (Lavelle and Sokol 2020) independently confirmed depleted microbial tryptophan catabolites across multiple IBD cohorts using complementary profiling approaches; the convergence of these findings across different analytical platforms strengthens confidence in the association, even though neither study design permits causal inference.

Beyond metabolomic associations, direct human tissue evidence supports the relevance of this axis in IBD. Monteleone et al. demonstrated that AhR expression is significantly reduced in intestinal mucosal tissue of IBD patients compared with healthy controls, and that stimulation of lamina propria mononuclear cells from IBD patients with an AhR agonist decreased IFN‐γ and upregulated IL‐22, providing direct ex vivo evidence that the AhR pathway remains functionally responsive in human IBD tissue despite its downregulation (Monteleone et al. 2011). The only available randomized controlled evidence of AhR‐targeted intervention in humans comes from the INDIGO trial, in which indigo naturalis, a preparation containing AhR ligands, demonstrated significant, dose‐dependent clinical response rates and mucosal healing in active UC, though the trial was terminated early due to pulmonary arterial hypertension risk, and the authors advised against clinical use pending further safety evaluation (Naganuma et al. 2018). While indigo naturalis is not a microbiota‐derived indole metabolite, this trial provides proof‐of‐concept that AhR activation can produce measurable clinical benefit in human IBD, supporting the rationale for developing more selective postbiotic AhR agonists.

The translational challenges of this field are further illustrated by the broader history of IBD drug development, in which multiple interventions showing robust efficacy in murine colitis models have subsequently failed in human trials, a pattern that underscores the need for caution in extrapolating preclinical AhR‐targeted findings to clinical predictions (Katsandegwaza et al. 2022; Valatas et al. 2013).

However, the strength of this evidence base is constrained by several methodological factors that limit how confidently these associations can be interpreted. Most human metabolomic data in IBD come from cross‐sectional cohorts, which can establish association but not causation or temporal sequence; it remains unclear whether reduced indole levels are a cause of barrier dysfunction or simply a consequence of dysbiosis, reduced dietary intake, or active inflammation itself (Kiran et al. 2026). Few studies have prospectively tracked indole metabolite trajectories alongside disease activity over time, and almost none have used interventional designs capable of testing whether restoring these metabolites changes clinical outcomes (Radhakrishnan et al. 2025). Quantification is further complicated by a lack of methodological standardization across studies. Differences in sample type (fecal, serum, or urine), collection and storage protocols, and analytical platforms (NMR, LC‐MS, or GC‐MS) introduce systematic variability that makes cross‐study comparisons difficult. This methodological heterogeneity may partly explain the inconsistent findings regarding which specific indole species are most strongly altered in IBD (Jagt et al. 2022; Vich Vila et al. 2024). In addition, diet, medication exposure (including biologics and antibiotics), and disease stage are known confounders of tryptophan and indole metabolite levels but are inconsistently controlled for across existing cohorts, raising the possibility that some reported associations partly reflect treatment or dietary differences between patients and controls rather than a primary defect in microbial tryptophan metabolism (Kiran et al. 2026; R. Chen et al. 2021).

No randomized interventional trials have directly tested microbiota‐derived purified indole metabolites, defined AhR‐ligand‐producing probiotic strains, or postbiotic formulations in IBD patients (Maftei et al. 2026). Further challenges include pharmacokinetic characterization of metabolites, safety evaluation, optimal delivery strategies, patient stratification based on microbiome and metabolome profiles, identification of predictive biomarkers, and integration with existing therapies.

Future work should prioritize prospective, longitudinal designs with standardized metabolomic methodology, early‐phase interventional trials of purified metabolites or defined probiotic strains, and mechanistic human studies that directly assess barrier function and AhR activation rather than relying on metabolite correlations alone. Until such data are available, targeting the microbial tryptophan‐AhR axis in IBD should be regarded as biologically promising but clinically unproven.

## 7 Conclusion

The gut microbiota‐tryptophan‐AhR axis functions not as a unidirectional pathway but as a dynamic, bidirectional network. Intestinal inflammation depletes the microbial taxa and substrate availability needed to sustain protective indole production, while the resulting loss of AhR ligands further compromises barrier integrity and perpetuates inflammation, creating a self‐reinforcing cycle that is impaired at multiple points simultaneously in IBD.

Nevertheless, the therapeutic relevance of this axis must be qualified. AhR activation is not uniformly beneficial, and its biological effects depend on both ligand specificity and the cellular and inflammatory context. The immunosuppressive or pro‐tumorigenic potential of sustained or kynurenine‐mediated receptor engagement underscores the need for ligand‐selective strategies that replicate the transient, low‐affinity kinetics of physiological indole metabolites rather than broadly amplifying AhR signaling.

The mechanistic evidence reviewed in this article demonstrates that microbial tryptophan metabolites coordinate multiple downstream pathways, AMPK, Nrf2, IL‐22, and IL‐10R signaling, to restore epithelial barrier function and attenuate intestinal inflammation, with postbiotic delivery offering practical advantages of dose standardization and stability over live bacterial interventions.

Although human data are still limited, reduced indole metabolite levels in IBD and supportive genetic and microbiome evidence strongly suggest translational relevance. Overall, targeting the microbial tryptophan‐AhR axis through ligand‐selective, physiologically informed postbiotic strategies represents a biologically compelling but clinically unproven approach; its translation into effective IBD therapy will depend on the conduct of well‐designed human interventional trials, standardized metabolomic methodology, and careful attention to ligand selectivity and safety.

## Author Contributions

**Mohammad Ali Mahdiabadi:** writing – review and editing, writing – original draft, investigation. **Asma Moghaddam:** data curation, formal analysis, writing – review and editing. **Nafiseh Erfanian:** conceptualization, supervision, writing – review and editing, project administration.

## Funding

The authors have nothing to report.

## Disclosure

The authors have nothing to report.

## Ethics Statement

The authors have nothing to report.

## Conflicts of Interest

The authors declare no conflicts of interest.

## Data Availability

The authors have nothing to report.
